# Highly Sensitive, Calibration-Free WM-DAS Method for Recovering Absorbance—Part II: Experimental Analysis

**DOI:** 10.3390/s20030616

**Published:** 2020-01-22

**Authors:** Zhimin Peng, Yanjun Du, Yanjun Ding

**Affiliations:** State Key Laboratory of Power Systems, Department of Energy and Power Engineering, Tsinghua University, Beijing 100084, China; apspect@tsinghua.edu.cn (Z.P.); dyj@tsinghua.edu.cn (Y.D.)

**Keywords:** tunable diode laser absorption spectroscopy, absorbance, fast-Fourier transform, calibration-free, gas temperature and concentration, spectroscopic parameters

## Abstract

Following the theoretical work in Part I, in this experimental study, the robustness, temporal resolution, and the narrow scan performance of the proposed wavelength modulation-direct absorption spectroscopy (WM-DAS) method are experimentally validated in a high-temperature tube furnace. The electromagnetic and other random-frequency noises can be effectively eliminated by extracting the characteristic spectra of the light intensity. The performance of WM-DAS with modulation frequencies from 0.1 to 100 kHz and scan indexes from 3.3 to 11.1 are also investigated at atmospheric pressure. The proposed method produces accurate line profile and high SNR over 500 consistently even with a weak absorption. As for real applications, the spectral line parameters of CO at 4300.6999 cm^−1^ including the collisional broadening, Dicke narrowing, and their dependence on temperature are measured. Furthermore, the high-speed measurement (1 ms) of the temperature and CO concentration of a McKenna flat flame are demonstrated.

## 1. Introduction

Through years of development, tunable diode laser absorption spectroscopy (TDLAS) has become one of the most important approaches in gas monitoring and has succeeded in various fields, including spectral line parameter measurements [1,2,3,4], and the diagnostic of combustion [5,6,7,8,9] and plasma [10,11,12]. Particularly, the 2*f*/1*f* method introduced by Hanson et al. [13,14,15] that overcomes the complicated calibration issue has further promoted the TDLAS technique toward practical applications. Meanwhile, Siemens, Aerodyne Research, and other companies have developed numerous online monitoring equipment based on TDLAS [16,17,18], including the LDS6 analyzer for NH_3_, CO, and other exhaust gases. In recent years, more demands for improved anti-noise ability, high signal-to-noise ratio (SNR) and high temporal resolution are put forward for TDLAS.

In the spectral line parameters measurement, to further increase the measurement accuracy, more and more attention is paid to some weak mechanisms, especially the line narrowing effect. The pronounced gull-wing signature in the best-fit Voigt profile residual and its disappearance in more advanced GP, RP, or SDVP profile fit suggest the non-negligible collisional narrowing effect [1,3,19,20]. In addition, as reported by David A. Long et al. [21], for the CO_2_ *R*16 transition at *P* = 6.7 kPa, the Lorentzian width and integrated area obtained through a Voigt profile fit which ignores the line narrowing effect are underestimated as much as 6% and 2%, respectively. However, the structured gull-wing residual is generally ~1% of the peak absorbance; that is to say, it can only be observed with an SNR of at least several hundreds. Moreover, the quantification of this narrowing effect has even higher requirements for the experiments. Except for some denoising algorithms [22], increasing the absorption by either extending the absorption length or turning to strong absorption lines is often used in most literature [2,20,23,24] as a compromising way of yielding high SNR. For example, Hanson et al. [25] measured the Dicke narrowing coefficients of several strong transitions of H_2_O near 1.4 μm using a gas cell as long as 76.2 cm to ensure a peak absorbance of ~10^−1^. However, limited by the temperature uniformity and confined space of the high-temperature furnace, the optical length is hard to be directly extended or magnified by multiple reflection or other cavity enhance technology. Therefore, further improving the sensitivity and SNR of the TDLAS measurement method is essential for the spectral line parameter measurement.

Similar to spectral line parameters measurement, applications such as diagnostic of combustion and industrial exhaust gases also demand more robustness to noise for TDLAS. For instance, in the widely investigated McKenna flat flame with an optical length of several centimeters, the absorption of high-concentration H_2_O in the NIR region is generally smaller than 10% and the absorption of the low-concentration OH and CO is even smaller. Thus, the accurate quantifications of the species concentration and flame temperature are quite challenging due to the small absorption and limited SNR. As reported by Steven et al. [26] with an optical length of 4.1 cm and a temperature of 2019 K, the peak absorption of CO on a laminar non-premixed methane flame at 4322.1 cm^−1^ is only 0.55%, which has a best-fit residual of 2.1 × 10^−4^ and an SNR of only 26. The OH transition at 6549.3 cm^−1^ has an even smaller absorption of 0.14%, at almost the same level of background noise. Therefore, most studies have to turn to the strong absorbed lines of H_2_O and CO_2_ in the MIR region to yield high enough SNR for the flame temperature measurement [27,28,29,30]. In addition, the instability of flame and the perturbation of gas flow and other noise also require the TDLAS methodology to be more robust. For the application of TDLAS in the industrial site, the robustness is even more crucial. Taking the ammonia slip measurement as an example, the combustion exhaust of the power plant boiler is quite hot (~600 K) and full of fly ash and other particulates. To monitor the trace amount (0–5 ppm) of ammonia slip in such an unfavorable environment, the TDLAS measurement method must have anti-noise capacity and high enough SNR.

In addition to the noise robustness, the temporal resolution and compatibility with a narrow scan of TDLAS are in higher demand for applications in transient and high-pressure conditions. Taking the fuel ignition process in a shock tube as an example, temperature and gas concentration vary dramatically in just a few milliseconds under pressure of ~1 MPa [31,32]. It, therefore, requires TDLAS to have a temporal resolution of higher than tens of kHz. Meanwhile, under such a high-pressure condition, the line broadening would be much larger and wavelength scan or modulation index would be restricted by the limited laser scan range.

Considering the importance of the noise rejection ability, SNR and the temporal resolution of TDLAS, in Part I [33] we proposed a WM-DAS method based on the FFT analysis of a sine wave scan. This method possesses the advantages of both DAS and WMS, allowing the direct measurement on the absorbance and further gas properties. The analysis in Part I [33] theoretically demonstrated that enhanced SNR and noise robustness can be obtained by extracting the characteristic spectra of the transmitted light intensity. In addition, the accuracy of the proposed method is improved by using a more accurate description of laser FM and IM response as well as the simultaneous fitting algorithm for the baseline and absorbance. In Part II, the experimental investigation of the proposed WM-DAS method will be presented. The characteristic of the proposed WM-DAS including the robustness, temporal resolution, and the narrow scan performance is experimentally validated with the CO transition in a high-temperature tube furnace. By extracting the characteristic spectra in the FFT analysis, the electromagnetic and other random-frequency noises are effectively eliminated. The performance of the proposed WM-DAS with modulation frequencies from 0.1 to 100 kHz and scan factors from 3.3 to 11.1 are further experimentally investigated. Both the accordance of the inferred spectral parameters and the SNR are taken as indicators to evaluate the performance. At last, as real experimental applications, the proposed WM-DAS is employed to measure the spectral line parameters of CO at 4300.6999 cm^−1^ including the collisional broadening, Dicke narrowing, and their dependence on temperature. Furthermore, the high-speed measurement (1 ms) of the temperature and CO concentration of a McKenna flat flame are further investigated with the proposed method.

## 2. Experimental System

**Figure 1:**
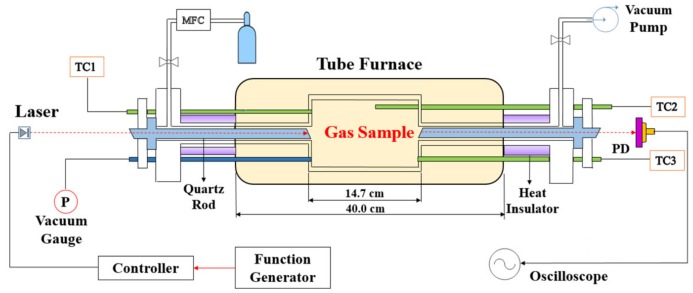
Schematic diagram of the measurement in the high-temperature tube furnace.

In this paper, both static high-temperature tube furnace and flat-flame burner were used to experimentally investigate the performance of the proposed WM-DAS method. For the high-temperature tube furnace, instead of the commonly used three-zone quartz optical cell [1,4,34,35] which has good non-absorbability to polarized molecular but poor impermeability, a self-designed gas cell was used as shown in Figure 1. The main body of the cell was made of 316 L stainless steel with a polished, coated inner surface to maintain both impermeability and non-absorbability. Two fused silica rods (45 cm) with flanges were inserted into both ends of the cell to construct a uniform test section. The heated cell has a volume of 498 cm^3^ and the gas leak rate is only 0.02 Pa/min at high vacuum condition. Three type B thermocouples (TC1, TC2, TC3) with an accuracy of 0.25% were mounted at equal spacing along the central zone of the cell. The maximum temperature gradient along the 14.7 cm test section was 3 K with a temperature of 1000 K measured by the three thermocouples. Other details are included in [23]. This customized system has the advantages of: (1) the unwanted absorption in the side zones with a large temperature gradient was effectively eliminated by the filled quartz rods; (2) the gas cell was sealed by O-rings and flanges exposed to the atmospheric environment, which enhances gas impermeability to a standard gas leak rate of 0.02 Pa/min under a high vacuum; (3) the pressure-resistant stainless steel cell and quartz rods make it possible to work under pressure as high as ~2 MPa.

**Figure 2:**
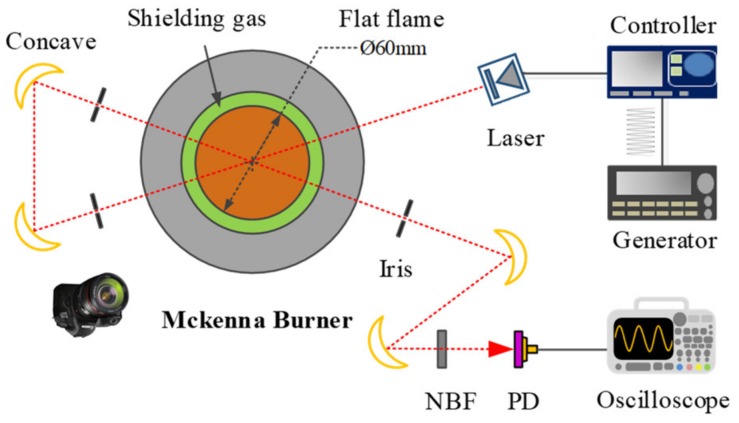
Schematic of the CO measurement on a McKenna burner. NBP: narrow band filter.

The schematic diagram of the McKenna burner is shown in Figure 2. A premixed CH_4_/air flame is established above a porous sintered burner plate with a diameter of 6 cm. The flame is stabilized and shielded by the dry compressed air co-flow coming from the outer shroud ring to eliminate the ambient interference, and the dry air flow rate is maintained at 40~50 L/min. The CH_4_ (1.0–1.5 L/min) and air (10–15 L/min) were controlled by two MKS mass flow controllers and mixed before sent to the burner. The burner is placed on a lab jack so that the laser can go through the flame at different heights above the burner (HAB). The laser went through the center of the axisymmetric flame twice to double the absorption length. Two pinholes with a diameter of 1.5 mm were placed in the light pass to minimize the intense background radiation from the flame. Meanwhile, the flame was recorded vertically by a camera to decide the effective optical length for different HABs.

In both experiments, the laser current is modulated by a sine waveform produced by a function generator (Keysight 33,500 B). The CO lines used in this paper are listed in Table 1. It is worth noticing that the energy level of Line 2 is too high (~5100 cm^−1^) to be observed with a temperature lower than 1000 K. However, in high-temperature flames (>1500 K) the absorption of Line 2 is comparable with that of the low energy Line 1, which makes this line pair especially suitable for the temperature and CO concentration measurement.

## 3. Characteristic of WM-DAS

In this part, the robustness, temporal resolution, and the capability with a narrow scan of the proposed WM-DAS method will be validated through experiments in the static high-temperature tube furnace. In addition to the accuracy of the fitting parameters, the SNR of the fitting is also used as an important indicator to evaluate the performance of the method. Here we define the SNR as follows for clarity,

(1) $$SNR=\frac{\alpha {\left(v\right)}_{\max}}{\sigma_{\mathrm{std}}},$$

where α(*v*)_max_ and *σ*_std_ represent the peak absorbance and the standard deviation of the best-fit residual, respectively.

### 3.1. Noise Rejection Ability

As is known to us, TDLAS measurement can be potentially disturbed by kinds of noises—including electromagnetic, vibration, and particle noise—which are difficult to be avoided even in an ideal laboratorial condition. Hence, a noise-insusceptible TDLAS process is in great demand, especially for the in situ industrial applications.

**Figure 3:**
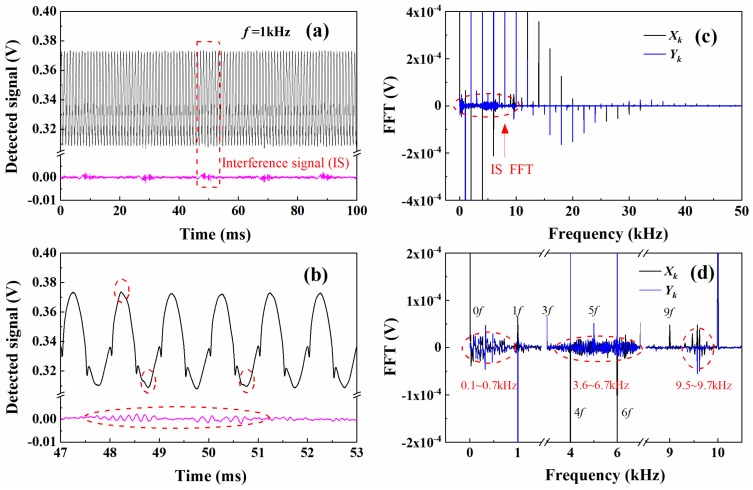
(**a**,**b**) detected transmitted light intensity with noise, (**c**,**d**) its Fourier coefficients. The experimental condition is similar with that in Part I: CO/N_2_ mixture gas, *T* = 299 K, *L* = 14.7 cm, *Xco* = 1.02%, *P* = 101.3 kPa [33].

Figure 3a shows the detected transmitted light signal measured in a high-temperature tube furnace as shown in Figure 1 for the spectral line measurement, in which the noise signal can be readily identified by the blue line. The noise consists of a typical 50 Hz electromagnetic perturbation, which can be eliminated by deliberately shielding the detecting system, as well as other high-frequency components. To demonstrate the behavior of the WM-DAS method under noise, here the noise-included signal in Figure 3a was further analyzed. The laser scan rate used in the measurement was 1 kHz, and 100 periods are acquired for the analysis. As the zoom-in plot in Figure 3b, the noise peak is about 4 mV, more than 1% of the averaged transmitted laser intensity (340 mV), which obviously distorted the transmitted signal as marked by the red circles. Figure 3c shows the spectrum of the detected signal derived from FFT. As discussed in Equation (9) from Part I, [33] theoretically, the spectrum of transmitted intensity distributes only at the integer multiples of the modulation frequency, i.e., *kf* (*k* = 0, 1, 2 …). However, in the actual measurement result, the noise signal not only locates at *kf*, but also spreads over the entire frequency range. As shown in Figure 3d, the noise frequency spectrum is distinctly observed at 0.1–0.7, 3.6–6.7, and 9.5–9.7 kHz. Despite the existence of these noise signals, the WM-DAS method extracts only the FFT coefficients at *kf* (*k* = 0, 1, 2 …) frequencies and thus eliminates the disturbance of noise to a great extent.

**Figure 4:**
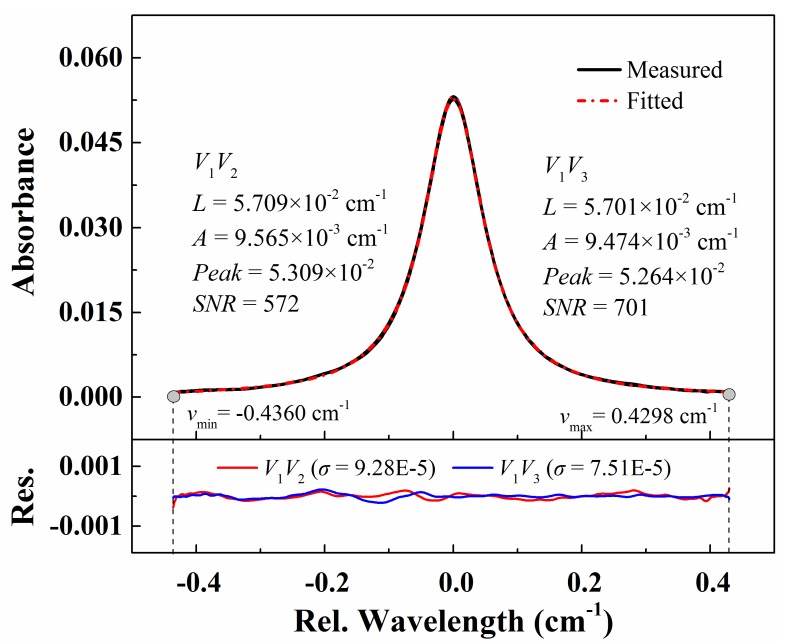
Recovered and best-fit absorbance from the detected signal with noise. The experimental conditions are: CO/N_2_ mixture gas, *T* = 299 K, *L* = 14.7 cm, *Xco* = 1.02%, *P* = 101.3 kPa.

By substituting the FFT coefficients at *kf* (*k* = 0, 1, 2 …) frequencies into the recovery formula and combining other processes in Part I [33] including the time-frequency transformation and simultaneous fitting, the absorbance obtained from *V*_1_*V*_2_ and *V*_1_*V*_3_ edges are shown in Figure 4. The best-fit parameters including the collisional broadening widths, the integrated absorbance and the peak absorbance for both edges are also listed in Figure 4. The inferred collisional broadening widths and integrated absorbance agreed within 0.6% and 1.2% of those from the noise-free experiment in Part I (Figure 7) [33], which reveals the good noise rejection ability of the proposed WM-DAS method. In addition, the best-fit residual is one order of magnitude smaller than that in [20] with traditional DAS, and the accordance of the inferred collisional broadening widths and integrated absorbance for both sides is also better than that in [20]. It must be admitted that although most noise can be eliminated, the noise signals at exactly *kf* frequencies will sneak through the process. Therefore, the scan rate should be optimized in practice to minimize the overlapping of noise signals at *kf* (*k* = 0, 1, 2 …) frequencies and thus enhance the SNR and precision of absorbance measurement.

### 3.2. Temporal Resolution

**Figure 5:**
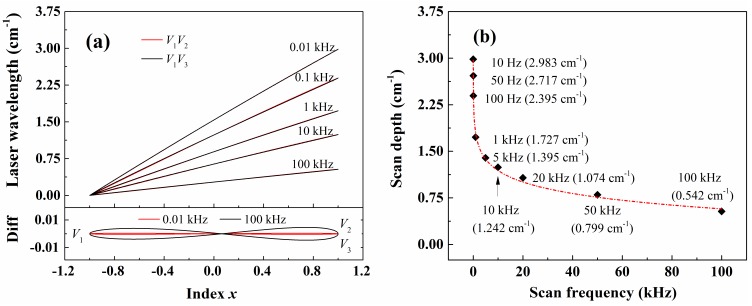
(**a**) The relationship between laser wavelength and variable *x* in both rising (*V*_1_*V*_3_) and falling (*V*_1_*V*_2_) edges for different modulation frequencies. (**b**) The relationship between the tuning coefficient and modulation frequency.

To analyze the temporal resolution of the WM-DAS method, experiments with different scan rates (0.01–100 kHz) were conducted in a high-temperature tube furnace as shown in Figure 1. As a revelation of the behavior of laser FM, the laser frequency responses with different scan rates were measured with the same current modulation amplitude (± 20 mA @ 140 mA). Figure 5a plots the laser wavelength in both *V*_1_*V*_2_ and *V*_1_*V*_3_ edges versus variable *x* with different scan rates. As can be seen from the bottom panel which shows the wavelength deviations from its average value of both edges, the wavelength difference between *V*_1_*V*_2_ and *V*_1_*V*_3_ is 0.5% of the total scan width with *f* = 100 kHz, while it is only 0.05% when the scan rate is 0.01 kHz. This suggests that the non-linearity of the laser frequency becomes more significant as the scan rate increases. Besides, a plot of the total scan range versus scan rate, as shown in Figure 5b, indicates that the laser wavelength scan range decreases exponentially with increasing scan rate [36]. For example, the scan range is 2.983 cm^−1^ (current coefficient 0.0746 cm^−1^/mA @ 140 mA) when the scan rate is 0.01 kHz whereas the scan range reduces to 0.542 cm^−1^ (current coefficient 0.0136 cm^−1^/mA @ 140 mA) when at 100 kHz scan rate. Therefore, a larger scan current is needed to cover the entire line shape with a high-frequency scan.

**Figure 6:**
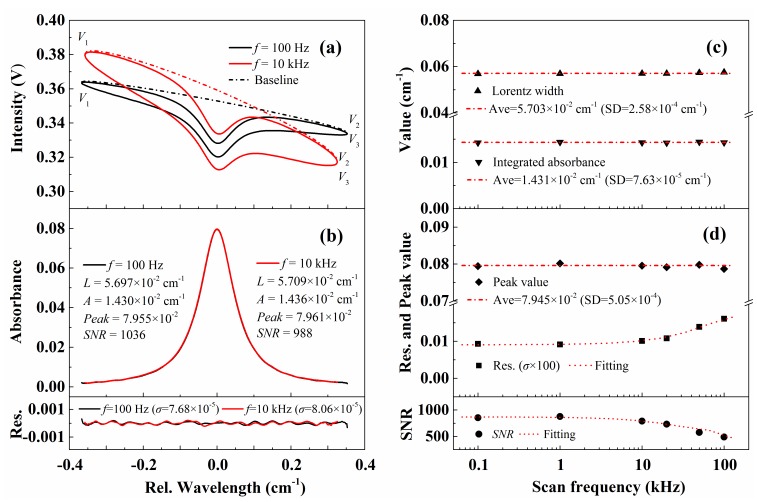
The recovered light intensity (**a**) and the best-fit absorbance (**b**) with different scan frequencies of 100 Hz and 10 kHz. (**c**,**d**) The best-fit parameters including the Lorentz width, integrated area, peak value, standard deviation of residual and SNR with different scan frequencies. The experimental conditions are: CO/N_2_ mixture gas, *T* = 299 K, *L* = 14.7 cm, *X*co = 1.53%, *P* = 100.6 kPa.

**Figure 7:**
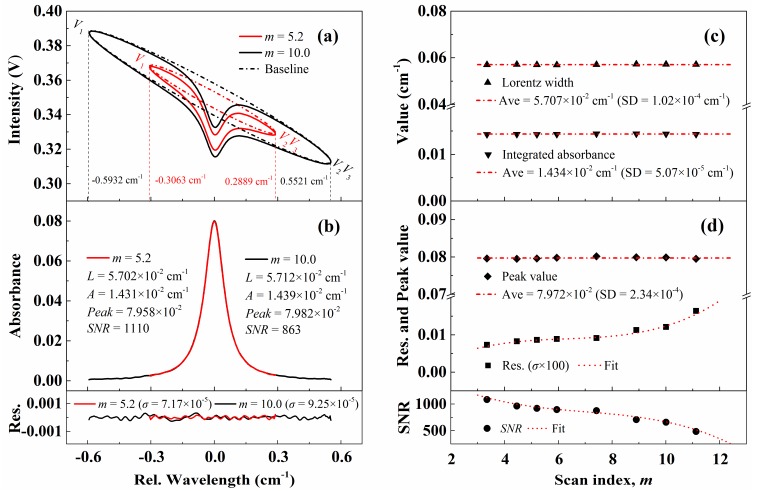
Recovered light intensity (**a**) and the best-fit absorbance (**b**) with scan indexes of 5.3 and 10. (**c**,**d**) The best-fit parameters including the Lorentz width, integrated area, peak value, standard deviation of residual and SNR with different scan indexes. The experimental conditions are: CO/N_2_ mixture gas, *T* = 299 K, *L* = 14.7 cm, *Xco* = 1.53%, *P* = 100.6 kPa.

Figure 6a,b illustrate the recovered light intensities and the best-fit absorbance with a scan frequency of 0.1 kHz and 10 kHz. The current scan amplitude was increased from ± 6 mA at 0.1 kHz to ± 11 mA at 10 kHz to keep an equivalent scan range of around 0.7 cm^−1^. The experiments were conducted with a pressure of 100.6 ± 0.01 kPa and a temperature of 299 ± 0.1 K. In Figure 6a, the ellipse-like structure becomes evident with high scan frequency. This is mainly caused by the large phase difference between FM and IM as shown in Equation (14) in Part I [33]. Using the recovered transmitted intensity and the simultaneous recovery algorithm for absorbance and baseline from Part I [33], the absorbance can be obtained, as shown in Figure 6b. As can be seen, the inferred collisional widths, integrated areas and peak absorption with scan rates of 0.1 kHz and 10 kHz agree very well with each other with deviations of 0.2%, 0.5%, and 0.1%, respectively. Meanwhile, the average collisional broadening width (5.703 × 10^−2^ cm^−1^) in this paper are 0.7% smaller than that from Part I (Figure 7, 5.741 × 10^−2^ cm^−1^) [33]. This may result from the 0.7% smaller working pressure in this paper (100.6 kPa) compared with that in Part I (101.3 kPa) [33], which also suggests the high sensitivity of the proposed method.

Furthermore, the absorbance under the same condition is measured at different scan frequencies from 0.1 kHz to 100 kHz. The best-fit collisional width, integrated absorbance, peak value, fitting residual and SNR are shown in Figure 6c,d. The best-fit Lorentz widths and integrated areas keep consistent over the measured scan frequency range with a relative standard deviation smaller than 0.5%. We noticed that the standard deviation of the best-fit residual of the recovered absorbance increases with high scan frequency, and thus the SNR decreases accordingly. This is mainly due to the following two reasons: (1) the non-linearity of laser FM is more significant under high scan rate, causing more uncertainties in determining its real wavelength; (2) larger current scan amplitude to ensure an equivalent wavelength scan range inevitably results in a larger intensity modulation. Considering the limited vertical resolution of the data acquisition system and the non-linear response of the photoelectric detector, the standard deviation of the best-fit residual would also be larger. In fact, if the laser intensity is stabilized (constant during the wavelength modulation), the SNR of the absorbance measurement would be much higher [37].

### 3.3. Performance with Narrow Scan

A large enough wavelength scan range is always required in DAS to ensure the ‘non-absorption’ zones for the baseline fitting. However, this demand always conflicts against the restricted operation current range and thus the limited wavelength tuning range of the laser. On one hand, as observed in Figure 5b, the laser tuning coefficient (wavelength scan range per current scan amplitude) drops drastically with the modulation frequency (*f* < 100 kHz) at a specific bias current [36,38,39]. That means, with a high scan frequency, a larger current modulation amplitude is required to achieve a sufficient scan range. On the other hand, under some high-pressure conditions where the collisional width is much larger and the line overlapping is severe, the non-absorption region is even more difficult to be ensured. Therefore, it is essential to check the performance of the proposed WM-DAS method with a narrow scan range. Here we define the scan index as *m* = scan depth/Δ*v*, where Δ*v* is the FWHM of the target absorption line.

Figure 7a,b compare the recovered light intensity and the best-fit absorbance with scan indexes of 5.2 and 10.0. The experimental temperature, pressure, and CO concentration are identical to the experiments described in Figure 6 and the laser scan rate is fixed at 1 kHz. It is clear that the recovered light intensities with the same modulation frequency (*f* = 1 kHz) but different scanning indexes present a similar structure, while it is not the case for those with different scan rates as shown in Figure 6. This is caused by the strong dependence of phase difference between laser FM and IM on the scan rate rather than the scan amplitude. The best-fit absorbance for these two conditions is shown in Figure 7b. As shown by the red line, with a scanning index of 5.2, the absorption at the endpoints *V*_1_ and *V*_2_ (*V*_3_) is still too strong to be ignored, which is far from the ‘non-absorption zones’. Even though, the inferred collisional width, integrated absorbance, and the peak absorbance from WM-DAS method agree well with the results in Figure 6 with a maximum relative difference of less than 0.2%.

Furthermore, measurements with different scanning indexes (*m* = 3.3~11.1) and a fixed laser scan rate (*f* = 1 kHz) are conducted and the results are exhibited in Figure 7c,d. It is obvious that the recovered absorbance remains accurate even with scan index as small as 3.3, which is clearly out of the implementation range for DAS. The standard deviations of collisional width, integrated area and peak absorbance for different scan indexes are 0.18%, 0.35%, and 0.29% of the corresponding average values. The consistency of measurement results with different scan indexes is better than that with different scan rates, and this may result from the relatively lower scan rate 1 kHz used in Figure 7, where the non-linearity effect is less significant. In addition, the SNR of measured absorbance decreased slightly with the increasing scan index, which has a similar reason with that in Figure 6. Despite the declines, all the SNRs for different scan rates and indexes in Figure 6 and Figure 7 are higher than 500, which ensures the accuracy of the measurement.

## 4. Applications of WM-DAS

As demonstrated in the previous section, the robustness, capabilities with high scan frequency and narrow scan range of the proposed WM-DAS method were experimentally validated in a static tube furnace. The good performance makes WM-DAS a great potential candidate for the complex actual applications. Therefore, in this section, this method was further applied to the high-accuracy measurement of spectroscopic parameters and the diagnostic on combustion specifically on a McKenna burner.

### 4.1. High-Accuracy Measurement of Spectroscopic Parameters (Experimental Scheme Shown in Figure 1)

**Figure 8:**
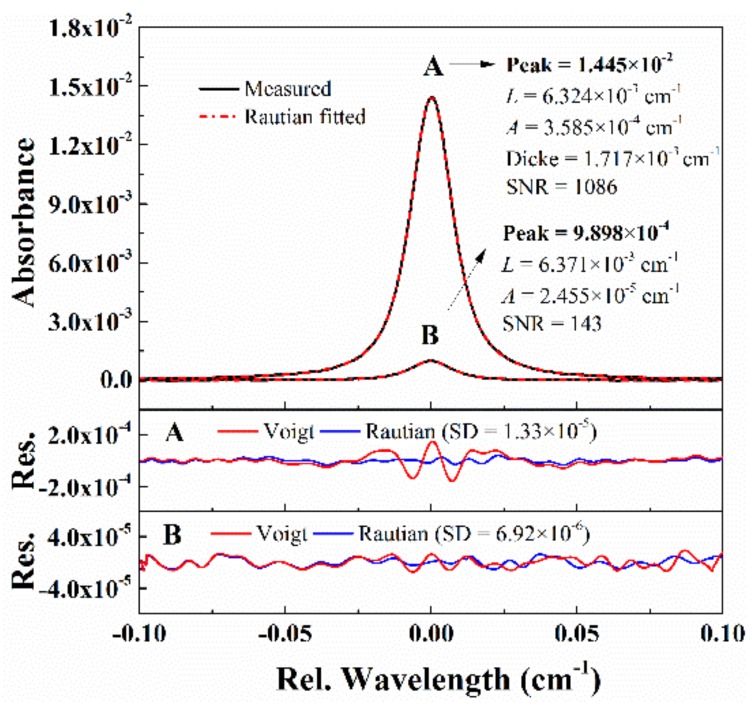
Examples of the recovered absorbance from WM-DAS method under pressure of 11.2 kPa and temperature of 299 K.

In the spectral line parameter measurement, SNR is of great importance for the quantification of some weak mechanisms, such as the Dicke narrowing. Figure 8 shows the absorbance of CO transition at 4300.6999 cm^−1^ measured with the WM-DAS method at 11.2 ± 0.01 kPa. In case A, when the peak absorbance is only 1.445 × 10^−2^, the SNR is still as high as 1086. The pronounced *w*-shaped residual is observed with Voigt fitting and disappears when the Rautian profile is used, which is vital for the quantification of the narrowing mechanism. The Dicke narrowing is obtained (1.717 × 10^−3^ cm^−1^) through the best-fit Rautian profile fitting. To the authors’ best knowledge, the evident observation and determination of narrowing effect under such low absorption conditions are reported for the first time. Further reducing the peak absorption to less than 0.1%, as shown in case B, the SNR is still as large as 143, which reflects the advantage of the proposed method in the spectral line parameter measurement.

**Figure 9:**
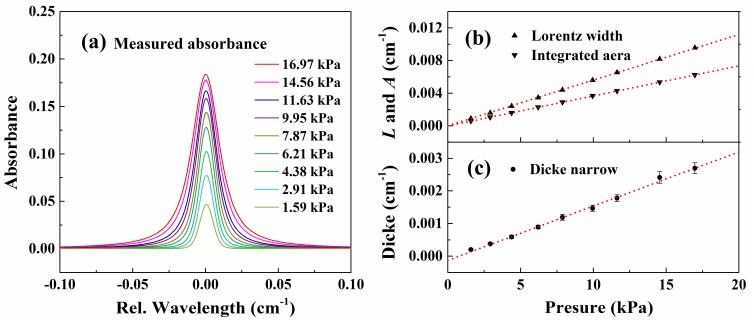
(**a**) Measured absorbance under different pressures. (**b**) Best-fit Lorentz widths and integrated areas versus pressure. (**c**) Best-fit Dicke narrowing coefficient and its two-parameter linear fit. The experimental conditions are: CO/N_2_ mixture gas, *T* = 299 K, *L* = 14.7 cm, *Xco* = 3.80%.

To eliminate the measurement uncertainty in the spectral line parameters under one certain pressure condition, the collisional broadening and Dicke narrowing coefficients are measured with different pressures ranging from 1.66 ± 0.01 kPa to 17.04 ± 0.01 kPa at *T* = 299 ± 0.1 K. Figure 9a shows a series of absorbance measured by WM-DAS under varying pressures using Rautian line shape fit with a fixed Gaussian width 5.03 × 10^−3^ cm^−1^. The SNRs for all pressures are in the range from 2000 to 3500. The measured collisional widths and integrated areas versus pressure are shown in Figure 9b, where the red dash line represents the best linear fitting. The *R*^2^ of the linear fit of collisional widths and integrated areas are larger than 0.99997 and the intercepts are smaller than 60 Pa, indicating perfect proportional behavior of the two parameters. The collisional broadening coefficient inferred from the slope of the linear fit is 5.739 × 10^−2^ (0.3%) cm^−1^/atm @ 299 K. The uncertainties in this section were estimated in a similar way as Nwaboh et al. [40]. In contrast to the Lorentz width, the measured Dicke narrowing has larger uncertainty as it is much smaller than the former one, as shown by the error bars in Figure 7c. In the pressure range of 7–12 kPa, where the Gauss and Lorentz widths are comparable, the measured Dicke narrowing coefficient is more accurate and reliable. From the slope of the linear fit with results in this pressure range, the measured Dicke narrowing coefficient is 1.7 × 10^−2^ (3.6%) cm^−1^/atm.

**Figure 10:**
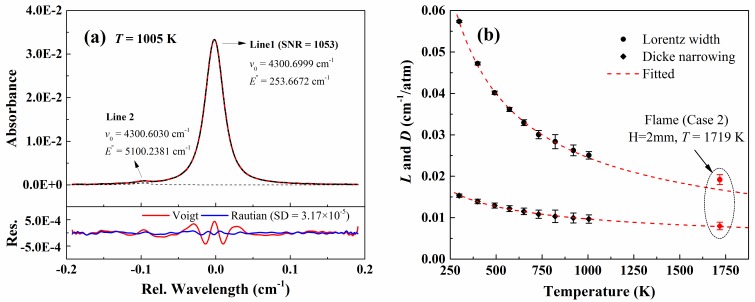
(**a**) Measured absorbance at a high temperature of 1005 K (*P* = 40.95 kPa, *L* = 14.7 cm). (**b**) Measured collisional width and Dicke narrowing and their corresponding best-fit power-law, for example, *L*(*T*) = *L*(*T*_0_)*(*T*_0_/*T*)^n^, used to determine temperature exponents *n*.

Experiments were also conducted at different temperatures from 299 K to 1005 K to obtain the collisional broadening coefficient *γ*_CO-N2_ (296 K), Dicke narrowing coefficient *β*_CO-N2_ (296 K) and their corresponding temperature exponents *n*. Figure 10a shows the measured absorbance of CO near 4300.7 cm^−1^ at 1005 K. It must be mentioned that the CO transition at 4300.6030 cm^−1^, which has an energy level as high as 5100 cm^−1^ marked as Line 2 in Table 1, must be taken into consideration in the fitting program under high-temperature condition. This peak will be more distinct at high temperatures and will be made full use of in the following section of the combustion diagnostic. Figure 10b plots the measured collisional broadening and Dicke narrowing together with their best-fit power-law used to determine *γ*_CO-N2_ (296 K) and *β*_CO-N2_ (296 K) and their respective temperature exponents *n*. 

From the best power-law fit, the collisional broadening coefficient γ_CO-N2_ at 296 K predicted by the intercept is 0.0577 (0.6%) cm^−1^/atm. As expected, the inferred value is 2% larger than that from HITRAN 2016 (0.0564 cm^−1^/atm), as mentioned in Part I [33]. Similar deviation has also been observed by others [19,20]. The temperature dependence of collisional broadening coefficient is 0.70 (0.6%), which is slightly smaller than that from HITRAN2016: 0.77 (<1%). The deviations in the collisional broadening coefficient and its temperature exponent may result from the different line profiles used in this paper (Rautian) and literature [41] (Voigt). Similar deviations have also been observed in the literature [23,25,42]. In addition, the relatively lower and narrower test temperature range (from 150 K to 298 K) in literature [41] may also contributes to the difference in the measured exponent. If only the first four low-temperature points (296–572 K) shown in Figure 10b are fitted with the power-law, the temperature dependence exponent would be 0.72, which is more closed to the results in [41]. This is also consistent with the phenomena that if the results from the higher-temperature combustion case, as shown by the red dot in Figure 10b, is considered in the fitting, the exponent would be even smaller, 0.64. Apparently, a broader temperature range will result in a more reliable temperature exponent although the uncertainty marked in literature [41] is incredibly small (<1%). Meanwhile, the temperature exponent of Dicke narrowing coefficient derived from the best-fit power-law curve in Figure 10b is 0.39, reported for the first time.

### 4.2. High-Speed Measurement on Combustion (Experimental Scheme Shown in Figure 2)

**Figure 11:**
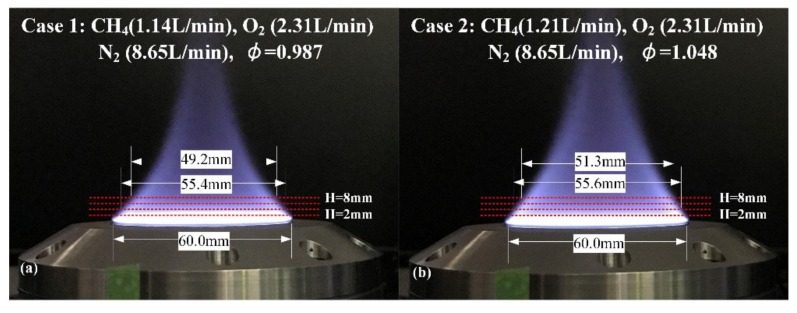
Images of the flame with different stoichiometric ratios. (**a**) *ϕ =* 0.987 (**b**) *ϕ =* 1.048.

The flat-flame is taken as an example of a transient object in this paper to verify the applicability and precision of WM-DAS method under the high-speed, dynamic conditions. Figure 11 shows the morphology of the flame with two different stoichiometric ratios of 0.98 and 1.05. The detailed flow rates of CH_4_, N_2_, and O_2_ are also listed in the corresponding figure. The temperatures and CO concentrations at HABs smaller than 10 mm where the CO absorptions are strong enough were investigated. The optical lengths at different HABs were determined from the flame images as shown by the red dashed line in Figure 11. It must be admitted that the uncertainty in the optical length contributes the most to the uncertainty in the final CO concentration. To eliminate the influence of the flame fluctuation on the measurement, the experiment was conducted with a high scan rate of 100 kHz.

**Figure 12:**
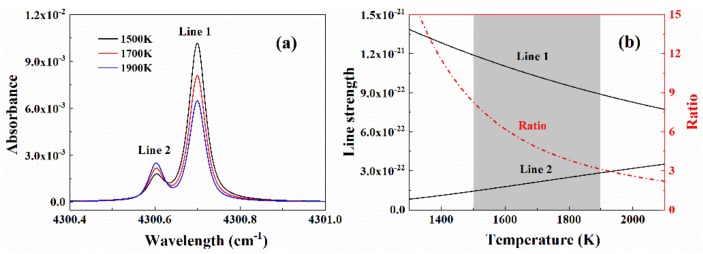
(**a**) Spectral simulation of the selected line pair of CO. (**b**) Line strengths and the ratio of selected line pair versus temperature. The simulation conditions are *P* = 1 atm, *L* = 11.56 cm, and 1.0% CO.

In a high-temperature environment such as the flame, the line pair of CO listed in Table 1 is very sensitive to temperature due to its large energy difference. Additionally, the small wavelength gap between these two lines (0.1 cm^−1^) allows them to be covered by one single laser scan. Therefore, this line pair is ideal for the high-speed measurement in the high-temperature flame. As an illustration of its temperature sensitivity, Figure 12a presents the simulated absorbance of these two lines under conditions similar to the investigated flame. As shown in Figure 12b, in the temperature range between 1500 K and 1900 K, both lines have sufficient absorption and the peak ratio of these two lines changes from 7 to 3, suggesting the good temperature sensitivity and measurement accuracy.

**Figure 13:**
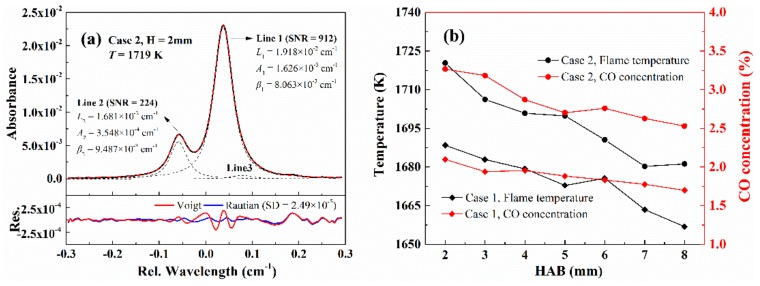
(**a**) An example of the recovered absorbance of CO in the CH_4_/Air flame together with the best-fit parameters. (**b**) The inferred flame temperature and CO concentraion for two cases.

Figure 13a shows the recovered CO absorption around 4300.7 cm^−1^ using the proposed WM-DAS method for case 2 at HAB = 2 mm. The black solid line represents the recovered absorbance and the red dash line is the best-fit overlapped absorbance that is composed of three individual lines marked as Lines 1–3. As can be seen from Figure 13a, the peak absorption of lines 1 and 2 are 2.3% and 0.6% with SNRs of 912 and 224, respectively. The best-fit residuals for both Voigt and Rautian profiles are also compared in the bottom panel of Figure 13a. In the best-fit Voigt profile residual, the *w*-shaped structure caused by the Dicke narrowing effect is pronounced around Line 1 and is buried in random noise for Line 2 due to its insufficient SNR. The best-fit Rautian profile effectively removes the *w*-shaped signature of Line 1 and reduces the standard deviation of residual by ~2.5 times compared to that of the Voigt profile. The best-fit collisional widths, integrated areas, and Dicke narrowing of both lines for this condition are also attached in Figure 13. The flame temperature was 1719 K deduced from the area ratio (*R* = 4.58 ± 0.037) and the CO concentration was 3.2% according to the inferred temperature and the integrated area of Line 1. In addition, the collisional width and Dicke narrowing for Line 1 for this high-temperature condition were also compared with the results from the previous section as shown in Figure 10. As marked by the red rhombus, the inferred Dicke-narrowing in this condition agreed within uncertainty with the predicted value. However, the collisional width is underestimated, as analyzed above, by the power-law curve fitted over the low-temperature domain. Similar to the analysis with HAB = 2 mm, the CO absorption at different HABs for two different stoichiometric ratios were further measured. Figure 13b compared the deduced CO concentrations and temperatures with the HAB in the range from 2 to 8 mm, beyond which the CO absorption is either severely disturbed by the flame or too weak to ensure a good SNR. It is clear that the CO concentration and flame temperature drops slightly with the increasing HAB and the decreasing stoichiometric ratio.

## 5. Conclusions

In this two-part paper, we proposed a calibration-free WM-DAS method based on the FFT analysis and the simultaneous fitting algorithm. As shown in the theoretical Part I [33], this method combines the advantages of measuring absorbance profile from calibration-free DAS with the enhanced noise rejection capacity and high sensitivity of WMS. In Part II, the robustness, the temporal resolution, and the performance with a narrow scan of the proposed method were experimentally validated using a high-temperature tube furnace. By extracting the characteristic frequency spectra of the WM-DAS after FFT analysis, noise from electromagnetic, vibration, and other sources were effectively screened in WM-DAS measurement. The SNR of the experiments with different peak absorptions were further investigated and the measurement SNR is more than 1000 with a weak absorption of ~1% using the purposed method. Then, the high-speed performance of WM-DAS was evaluated through a series of measurements with scan frequencies varying from 0.1 kHz to 100 kHz. The inferred Lorentz widths and integrated areas are consistent over the measured scan frequency range with a relative standard deviation smaller than 0.5%. In addition, the performance of the proposed WM-DAS with different scan widths was experimentally checked. Good accordance of the inferred collisional widths in the investigated scan range was achieved with a standard deviation of 1.02 × 10^−4^ cm^−1^, only 0.18% of the average value. Finally, as real applications, the spectral line parameters of CO at 4300.6999 cm^−1^ including the collisional broadening, Dicke narrowing and their dependence on temperature was measured using the proposed WM-DAS method. A more accurate collisional broadening coefficient *γ*_CO-N2_ and its temperature exponent were obtained with a Rautian profile fit and the Dicke narrowing coefficient and its temperature exponent were reported for the first time. Meanwhile, the CO concentration and the temperature profiles of a McKenna flat flame with different stoichiometric ratios were further investigated using the proposed method.

## Figures and Tables

**Table 1:** Line pair information for CO molecule near 4300 cm^−1^ used in this paper (296 K).

	*v*_0_ (cm^−1^)	*S* (cm^−2/^atm)	*γ*_ai__r_ (cm^−1^/atm)	*γ*_self_ (cm^−1^/atm)	*E*″ (cm^−1^)
Line1	4300.6999	2.616 × 10^−21^	0.0564	0.062	253.6672
Line2	4300.6030	1.935 × 10^−30^	0.0420	0.041	5100.2381

## References

[1] Goldenstein C.S., Jeffries J.B., Hanson R.K. (2013). Diode laser measurements of linestrength and temperature-dependent lineshape parameters of H_2_O-, CO_2_-, and N_2_-perturbed H_2_O transitions near 2474 and 2482 nm. J. Quant. Spectrosc. Radiat. Transf..

[2] Pogány A., Klein A., Ebert V. (2015). Measurement of water vapor line strengths in the 1.4–2.7 µm range by tunable diode laser absorption spectroscopy. J. Quant. Spectrosc. Radiat. Transf..

[3] Es-sebbar E., Farooq A. (2014). Intensities, broadening and narrowing parameters in the *v*_3_ band of methane. J. Quant. Spectrosc. Radiat. Transf..

[4] Li J.S., Liu N.W., Ding J.Y., Zhou S., He T.B., Zhang L. (2019). Piezoelectric effect-based detector for spectroscopic application. Opt. Lasers Eng..

[5] Allen M.G. (1998). Diode laser absorption sensors for gas-dynamic and combustion flows. Meas. Sci. Technol..

[6] Goldenstein C.S., Spearrin R.M., Jeffries J.B., Hanson R.K. (2017). Infrared laser-absorption sensing for combustion gases. Prog. Energy Combust. Sci..

[7] Xu K.K., Chen Y.X., Okhal T.A., Snyman L.W. (2019). Micro optical sensors based on avalanching silicon light-emitting devices monolithically integrated on chips. Opt. Mater Express.

[8] Seals L., Gole J.L., Tse L.A., Hesketh P.J. (2002). Rapid, reversible, sensitive porous silicon gas sensor. J. Appl. Phys..

[9] Hanson R.K. (2011). Applications of quantitative laser sensors to kinetics, propulsion and practical energy systems. Proc. Combust. Inst..

[10] Reuter S., Sousa J.S., Stancu G.D., van Helden J.P.H. (2015). Hubertus Van Helden. Review on VUV to MIR absorption spectroscopy of atmospheric pressure plasma jets. Plasma Sources Sci. Technol..

[11] Douat C., Kacem I., Sadeghi N., Bauville G., Fleury M., Puech V. (2016). Space-time resolved density of helium metastable atoms in a nanosecond pulsed plasma jet: Influence of high voltage and pulse frequency. J. Phys. D.

[12] Röpcke J., Lombardi G., Rousseau A., Davies P.B. (2006). Application of mid-infrared tunable diode laser absorption spectroscopy to plasma diagnostics: A review. Plasma Sources Sci. Technol..

[13] Goldenstein C.S., Strand C.L., Schultz I.A., Sun K., Jeffries J.B., Hanson R.K. (2014). Fitting of calibration-free scanned-wavelength-modulation spectroscopy spectra for determination of gas properties and absorption lineshapes. Appl. Opt..

[14] Rieker G.B., Jeffries J.B., Hanson R.K. (2009). Calibration-free wavelength-modulation spectroscopy for measurements of gas temperature and concentration in harsh environments. Appl. Opt..

[15] Sun K., Chao X., Sur R., Goldenstein C.S., Jeffries J.B., Hanson R.K. (2013). Analysis of calibration-free wavelength-scanned wavelength modulation spectroscopy for practical gas sensing using tunable diode lasers. Meas. Sci. Technol..

[16] McManus J.B., Zahniser M.S., Nelson J.D.D., Shorter J.H., Herndon S.C., Wood E.C., Wehr R. (2010). Application of quantum cascade lasers to high-precision atmospheric trace gas measurements. Opt. Eng..

[17] Nelson D.D., McManus J.B., Herndon S.C., Shorter J.H., Zahniser M.S., Blaser S., Hvozdara L., Muller A., Giovannini M., Faist J. (2006). Characterization of a near-room-temperature, continuous-wave quantum cascade laser for long-term, unattended monitoring of nitric oxide in the atmosphere. Opt. Lett..

[18] Xu K., Huang L., Zhang Z., Zhao J., Zhang Z., Snyman L.W., Swart J.W. (2018). Light emission from a poly-silicon device with carrier injection engineering. Mater. Sci. Eng. B.

[19] Mulvihill C.R., Alturaifi S.A., Petersen E.L. (2018). High-temperature He- and O_2_- broadening of the R(12) line in the 1←0 band of carbon monoxide. J. Quant. Spectrosc. Radiat. Transf..

[20] Du Y.J., Peng Z.M., Ding Y.J. (2018). High-accuracy sinewave-scanned direct absorption spectroscopy. Opt. Express.

[21] Long D.A., Bielska K., Lisak D., Havey D.K., Okumura M., Miller C.E., Hodges J.T. (2011). The air-broadened, near-infrared CO_2_ line shape in the spectrally isolated regime: Evidence of simultaneous Dicke narrowing and speed dependence. J. Chem. Phys..

[22] Zaharov V.V., Farahi R.H., Snyder P.J., Davision B.H., Passian A. (2014). Karhunen-Loeve treatment to remove noise and facilitate data analysis in sensing, spectroscopy and other applications. Analyst.

[23] Li J.D., Du Y.J., Peng Z.M., Ding Y.J. (2019). Measurements of spectroscopic parameters of CO_2_ transitions for Voigt, Rautian, Galatry and speed-dependent Voigt profiles near 1.43 µm using the WM-DAS method. J. Quant. Spectrosc. Radiat. Transf..

[24] McGettrick A.J., Duffin K., Johnstone W., Stewart G., Moodie D.G. (2008). Tunable diode laser spectroscopy with wavelength modulation: A phasor decomposition method for calibration-free measurements of gas concentration and pressure. J. Lightwave Technol..

[25] Li H., Farooq A., Jeffries J.B., Hanson R.K. (2008). Diode laser measurements of temperature-dependent collisional-narrowing and broadening parameters of Ar-perturbed H_2_O transitions at 1391.7 and 1397.8 nm. J. Quant. Spectrosc. Radiat. Transf..

[26] Buerkle S., Dreizler A., Ebert V., Wagner S. (2018). Experimental comparison of a 2D laminar diffusion flame under oxy-fuel and air atmosphere. Fuel.

[27] Li S., Farooq A., Hanson R.K. (2011). H_2_O temperature sensor for low-pressure flames using tunable diode laser absorption near 2.9 µm. Meas. Sci. Technol..

[28] Zheng S., Liang W.K., Chu H.Q., Zhou H.C. (2020). Effect of radiation reabsorption of C_1_-C_6_ hydrocarbon flames at normal and elevated pressures. Fuel.

[29] Liu C., Xu L., Cao Z. (2013). Measurement of nonuniform temperature and concentration distributions by combining line-of-sight tunable diode laser absorption spectroscopy with regularization methods. Appl. Opt..

[30] Ma L., Lau L., Ren W. (2017). Non-uniform temperature and species concentration measurements in a laminar flame using multi-band infrared absorption spectroscopy. Appl. Phys. B-Lasers O..

[31] Tranter R.S., Brezinsky K., Fulle D. (2001). Design of a high-pressure single pulse shock tube for chemical kinetic investigations. Rev. Sci. Instrum..

[32] Hanson R.K., Davidson D.F. (2014). Recent advances in laser absorption and shock tube methods for studies of combustion chemistry. Prog. Energy Combust. Sci..

[33] Peng Z., Du Y., Ding Y. (2020). Highly sensitive, Calibration-free WM-DAS method for recovering absolute absorbance—Part I: Theoretical analysis. Sensors.

[34] Tao B., Hu Z., Fan W., Wang S., Ye J., Zhang Z. (2017). Novel method for quantitative and real-time measurements on engine combustion at varying pressure based on the wavelength modulation spectroscopy. Opt. Express.

[35] Farooq A., Jeffries J.B., Hanson R.K. (2008). In situ combustion measurements of H_2_O and temperature near 2.5 µm using tunable diode laser absorption. Meas. Sci. Technol..

[36] Li H., Rieker G.B., Liu X., Jeffries J.B., Hanson R.K. (2006). Extension of wavelength-modulation spectroscopy to large modulation depth for diode laser absorption measurements in high-pressure gases. Appl. Opt..

[37] Zhao G., Tan W., Jia M., Hou J., Ma W., Dong L., Zhang L., Feng X., Wu X., Yin W. (2016). Intensity-stabilized fast-scanned direct absorption spectroscopy instrumentation based on a distributed feedback laser with detection sensitivity down to 4 × 10^−6^. Sensors.

[38] Benoy T., MLengden Stewart G., Johnstone W. (2016). Recovery of absorption line shapes with correction for the wavelength modulation characteristics of DFB lasers. IEEE Photonics J..

[39] Du Y., Peng Z.M., Ding Y.J. (2020). A high-accurate and universal method to characterize the relative wavelength response (RWR) in wavelength modulation spectroscopy (WMS). Opt. Express.

[40] Nwaboh J.A., Werhahn O., Ebert V. (2014). Line strength and collisional broadening coefficients of H_2_O at 2.7 µm for natural gas quality assurance applications. Mol. Phys..

[41] Devi V.M., Benner D.C., Smith M.A.H., Mantz A.W., Sung K., Brown L.R., Predoi-Cross A. (2012). Spectral line parameters including temperature dependences of self- and air-broadening in the 2←0 band of CO at 2.3 µm. J. Quant. Spectrosc. Radiat. Transf..

[42] Goldenstein C.S., Hanson R.K. (2015). Diode-laser measurements of linestrength and temperature-dependent lineshape parameters for H_2_O transitions near 1.4 µm using Voigt, Rautian, Galatry, and speed-dependent Voigt profiles. J. Quant. Spectrosc. Radiat. Transf..

